# The complete chloroplast genome of *Juniperus rigida* (Cupressaceae)

**DOI:** 10.1080/23802359.2022.2098858

**Published:** 2022-07-22

**Authors:** Mingcheng Wang, Rui Li

**Affiliations:** a Institute for Advanced Study, Chengdu University, Chengdu, China; b Engineering Research Center of Sichuan-Tibet Traditional Medicinal Plant, Chengdu, China; c School of Food and Biological Engineering, Chengdu University, Chengdu, China

**Keywords:** Chloroplast genome, Cupressaceae, *Juniperus rigida*, phylogenetic analyses

## Abstract

The complete chloroplast (cp) genome of *Juniperus rigida* was assembled using Illumina short reads. The assembled plastome showed a circular structure with an overall size of 127,720 bp. Inverted repeat (IRs) regions were absent from the *J. rigida* cp genome. A total of 119 genes were predicted, including 82 protein-coding genes, 33 tRNA genes, and 4 rRNA genes. The overall GC content of *J. rigida* cp genome was 34.92%. Phylogenetic analysis among *J. rigida* and 19 other Cupressaceae species showed that *J. rigida* clustered together with *J. formosana*. The *J. rigida* cp genome presented in this study will provide useful genetic resource for further evolutionary studies of the genus *Juniperus* as well as Cupressaceae.

*Juniperus rigida* Sieb. et Zucc. 1846 (Cupressaceae) is an evergreen shrub or a small tree that is mainly distributed in the cold temperate regions of Northern China, Korea, and Japan. In East Asian countries, the berries, branches, and leaves of *J. rigida* are utilized in traditional medicine to treat brucellosis, rheumatic arthritis, nephritis, edema, and skin disease (Feng et al. 2021). *J. rigida* is also planted as an ornamental and timber tree due to its lush green foliage and high wood quality (Orhan 2019). Despite its importance, the complete chloroplast (cp) genome of *J. rigida* has not been sequenced or reported to date, which hampers a deeper understanding of its genomic characterization and evolutionary history. In this study, we first assembled the cp genome of *J. rigida* and reconstructed a plastome phylogeny of the genus *Juniperus*.

Plant material collection was carried out in accordance with the Regulations of the People’s Republic of China on the Protection of Wild Plants. Fresh and mature leaves were collected from an adult plant of *J. rigida* growing in Alxa league, Inner Mongolia, China (38.9784°N, 105.9035°E). The voucher sample was deposited in the Herbarium of North Minzu University (Lei Zhang, zhangsanshi-0319@163.com, voucher number: NMU00078). Total genomic DNA was extracted using the CTAB method (Doyle and Doyle 1987). For Illumina sequencing, paired-end libraries with an insertion size of 350 bp were constructed in accordance with the manufacturer’s instructions and sequenced on an Illumina HiSeq 2500 platform. A total of 7.55 Gb Illumina data were generated. The cp genome of *J. rigida* was *de novo* assembled using NOVOPlasty (Dierckxsens et al. 2017). The assembled *J. rigida* cp genome was annotated using Plann (Huang and Cronk 2015) with the *J. squamata* cp genome (GenBank accession number: NC_044076) annotation as the reference, and the annotation was corrected using Geneious (Kearse et al. 2012).

The assembled cp genome of *J. rigida* (GenBank accession number: NC_062083) showed a circular structure with an overall size of 127,720 bp. Similar to other sequenced cp genomes of *Juniperus* species, inverted repeat (IR) regions were absent from the *J. rigida* cp genome. A total of 119 genes were predicted, including 82 protein-coding genes, 33 tRNA genes, and 4 rRNA genes. Among these, 9 protein-coding genes and 6 tRNA genes had one intron, and 1 protein-coding gene, *rps*12, had two introns. The overall GC content of *J. rigida* cp genome was 34.92%, and the GC content of genic regions (35.20%) was higher than that of the intergenic regions (33.59%). For protein-coding regions, the GC content of the first, second, and third codons was 45.53%, 36.07%, and 27.16%, respectively. The 82 protein-coding genes encoded a total of 25,284 codons, with leucine and cystine being the most (10.84%) and least (1.12%) frequently used amino acids, respectively.

Phylogenetic analysis was performed among *J. rigida* and 19 other Cupressaceae species, using *Platycladus orientalis* as outgroup. Coding sequences of 79 genes presented in all the 20 cp genomes of Cupressaceae species were aligned using the ‘linsi’ option of MAFFT v7.313 (Katoh and Standley 2013) and concatenated into a supermatrix. Finally, a maximum likelihood (ML) tree was constructed by RAxML v8.2.11 (Stamatakis 2006) with 500 bootstrap replicates based on the supermatrix, with the main parameters of ‘-f j -m GTRGAMMA.’ The resulting plastome phylogeny showed that *J. rigida* clustered together with *J. formosana* with 100% bootstrap support (Figure 1). The *J. rigida* cp genome presented in this study will provide useful genetic resource for further evolutionary studies of the genus *Juniperus* as well as Cupressaceae.

**Figure 1.**
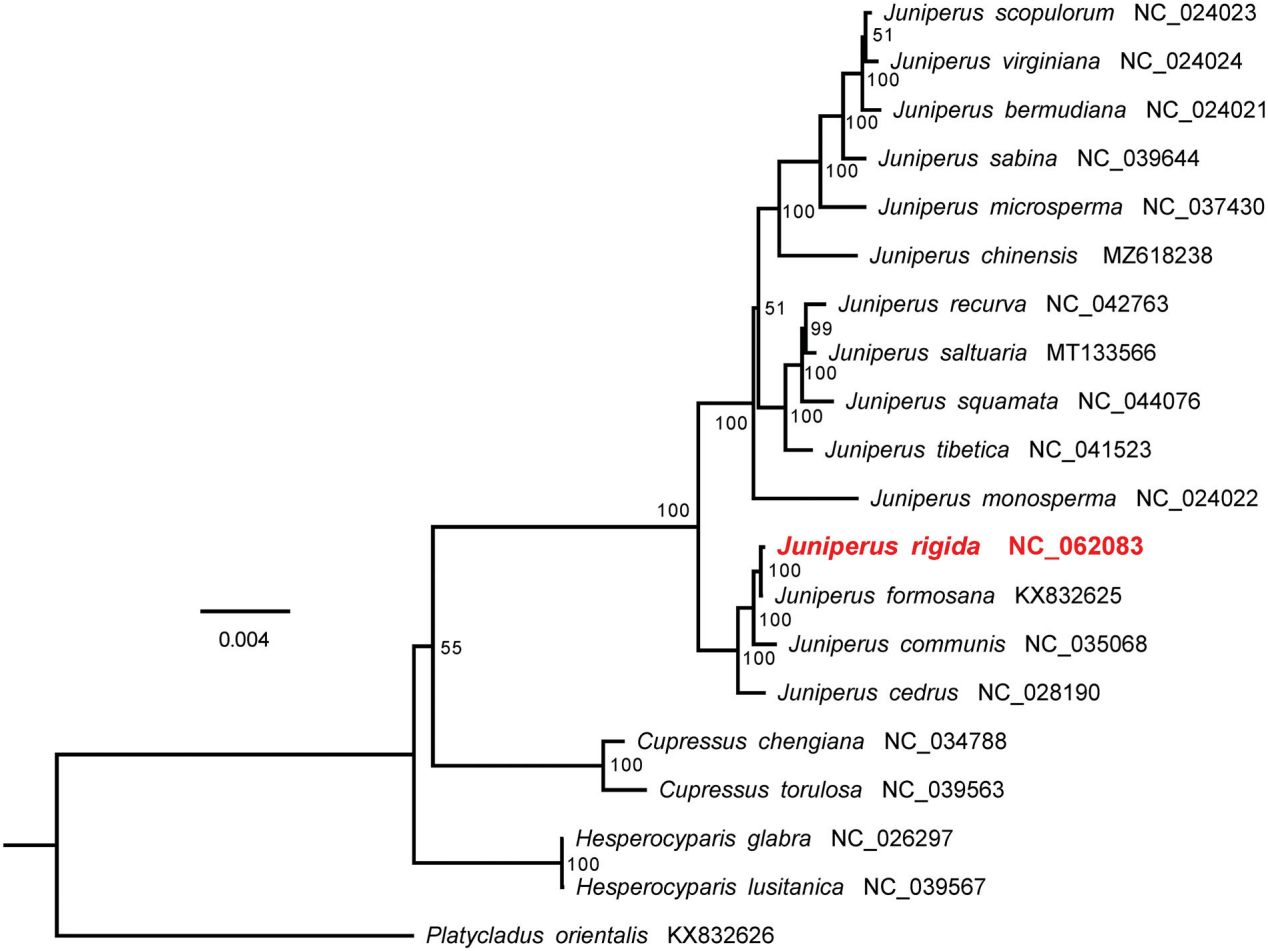
Maximum likelihood phylogeny based on coding sequences of 79 genes presented in all the 20 cp genomes of Cupressaceae species, with *Platycladus orientalis* as the outgroup. The number on each node indicates the bootstrap value.

## Data Availability

The genome sequence data that support the findings of this study are openly available in GenBank of NCBI at https://www.ncbi.nlm.nih.gov/, under the accession no. OM302213. The associated BioProject, SRA, and Bio-Sample numbers are PRJNA818779, SRR18439740, and SAMN26879214, respectively.
